# Resilience of the ruminal bacterial community to increasing inclusion of de-oiled wet distillers grains in feedlot diets

**DOI:** 10.1007/s11250-026-05242-z

**Published:** 2026-07-20

**Authors:** Gercino Ferreira Virginio Júnior, Pedro Henrique Francisco de Torres, Barbara Carolina Afonso, Bernardo Carvalho Petean, Johnny Maciel de Souza, Mario De Beni Arrigoni, Laís de Aquino Tomaz, Otávio Rodrigues Machado Neto, Welder Angelo Baldassini, Pablo de Souza Castagnino, Danilo Domingues Millen

**Affiliations:** 1 School of Agricultural and Veterinary Sciences, São Paulo State University (UNESP), Jaboticabal, São Paulo, Brazil; 2 School of Veterinary Medicine and Animal Science, São Paulo State University (UNESP), Botucatu, São Paulo, Brazil; 3 Federal University of Rio Grande do Sul, Porto Alegre, Rio Grande do Sul Brazil

**Keywords:** 16S rRNA gene amplicon sequencing, Alternative feed coproducts, Beef cattle nutrition, High-concentrate feeding systems, Microbial ecosystem stability

## Abstract

This study aimed to evaluate the impact of increasing de-oiled wet distillers grains (WDG) inclusion on the structure, diversity, and composition of the ruminal bacterial community in Nellore cattle fed high-concentrate diets. Four ruminally cannulated Nellore bulls were assigned to a 4 × 4 Latin square design and fed diets containing 0, 150, 300, or 450 g de-oiled WDG/kg dry matter. Ruminal samples were collected for bacterial DNA extraction and 16S rRNA gene amplicon sequencing to assess alpha diversity, beta diversity, and taxonomic composition. Increasing dietary inclusion of de-oiled WDG did not affect bacterial richness (*P* = 0.35) or overall community structure (*P* = 0.26). Permutational multivariate analysis of variance revealed no significant treatment effects on bacterial community structure (*P* > 0.05), indicating high microbial stability across diets. Relative abundance analysis showed that Firmicutes and Bacteroidetes remained the dominant phyla across all treatments, with no significant differences detected after multiple-testing correction (*P* > 0.05). Exploratory analyses identified a limited number of taxa contributing to between-treatment dissimilarities, mainly affiliated with *Firmicutes*, *Bacteroidetes*, *Verrucomicrobia*, and *Chloroflexi*; however, none of these differences remained significant after false discovery rate adjustment. Overall, increasing inclusion of de-oiled WDG in high-concentrate diets did not induce substantial shifts in the ruminal bacterial community. These findings suggest that the rumen microbiome of feedlot Nellore cattle is resilient to dietary replacement of conventional ingredients with de-oiled WDG up to 450 g/kg DM, supporting its practical application as a sustainable coproduct in intensive beef production systems without compromising microbial ecosystem stability.

**Supplementary Information:**

The online version contains supplementary material available at https://doi.org/10.1007/s11250-026-05242-z.

## Introduction

The rumen microbiome plays a central role in nutrient utilization and host performance in beef cattle. It is a highly dynamic and resilient ecosystem that adapts to dietary changes while maintaining functional stability (Flint et al. 2015; Weimer, 2015). However, changes in microbial composition do not always translate into measurable differences in animal performance or fermentation parameters, reflecting the functional redundancy of the ruminal ecosystem (Henderson et al. 2015; Weimer, 2015).

Distillers grains (DG) are widely used in feedlot diets due to their high energy content, rumen undegradable protein, and fiber concentration (Virgínio Júnior et al. 2026). Wet distillers grains (WDG) are commonly included at moderate to high levels as partial replacements for cereal grains and protein supplements (Luebbe et al. 2012; Baldassini et al. 2022). Increasing WDG inclusion alters dietary proportions of starch, fiber, lipids, and nitrogen, which may influence microbial metabolism and ecological niches in the rumen (Klopfenstein et al. 2008; Watson et al. 2014; Ferreira et al.^ 2020^).

The adoption of oil extraction technologies has increased the availability of de-oiled WDG, which contains lower ether extract and higher fiber concentrations than conventional WDG (Virgínio Júnior et al. 2026). This compositional shift may reduce lipid-associated inhibition of fibrolytic bacteria while maintaining the nutritional value of the coproduct. Despite these changes, the extent to which de-oiled WDG affects ruminal microbial community structure remains unclear.

Previous studies have focused primarily on intake, digestibility, and fermentation responses, reporting changes in short-chain fatty acid profiles without compromising ruminal pH or animal performance (Ferreira et al. 2020; Tomaz et al. 2021). These findings suggest that de-oiled WDG may alter microbial activity without necessarily inducing major shifts in community structure. However, direct evidence describing its effects on ruminal bacterial composition is still limited.

Therefore, this study evaluated the effects of increasing dietary inclusion of de-oiled WDG on ruminal bacterial communities in feedlot Nellore bulls. We hypothesized that increasing de-oiled WDG inclusion would promote subtle shifts in ruminal microbial composition while preserving overall community structure in a high-concentrate feeding system.

## Materials and methods

### Animals, experimental design, and diets

The experiment was conducted at the Animal Unit of Digestive and Metabolic Studies, Department of Animal Science, São Paulo State University (UNESP), Jaboticabal, São Paulo, Brazil. All experimental procedures were approved by the Animal Use Ethics Committee of the College of Veterinary Medicine and Animal Science – UNESP (protocol no. 00719711).

**Fig. 1:**
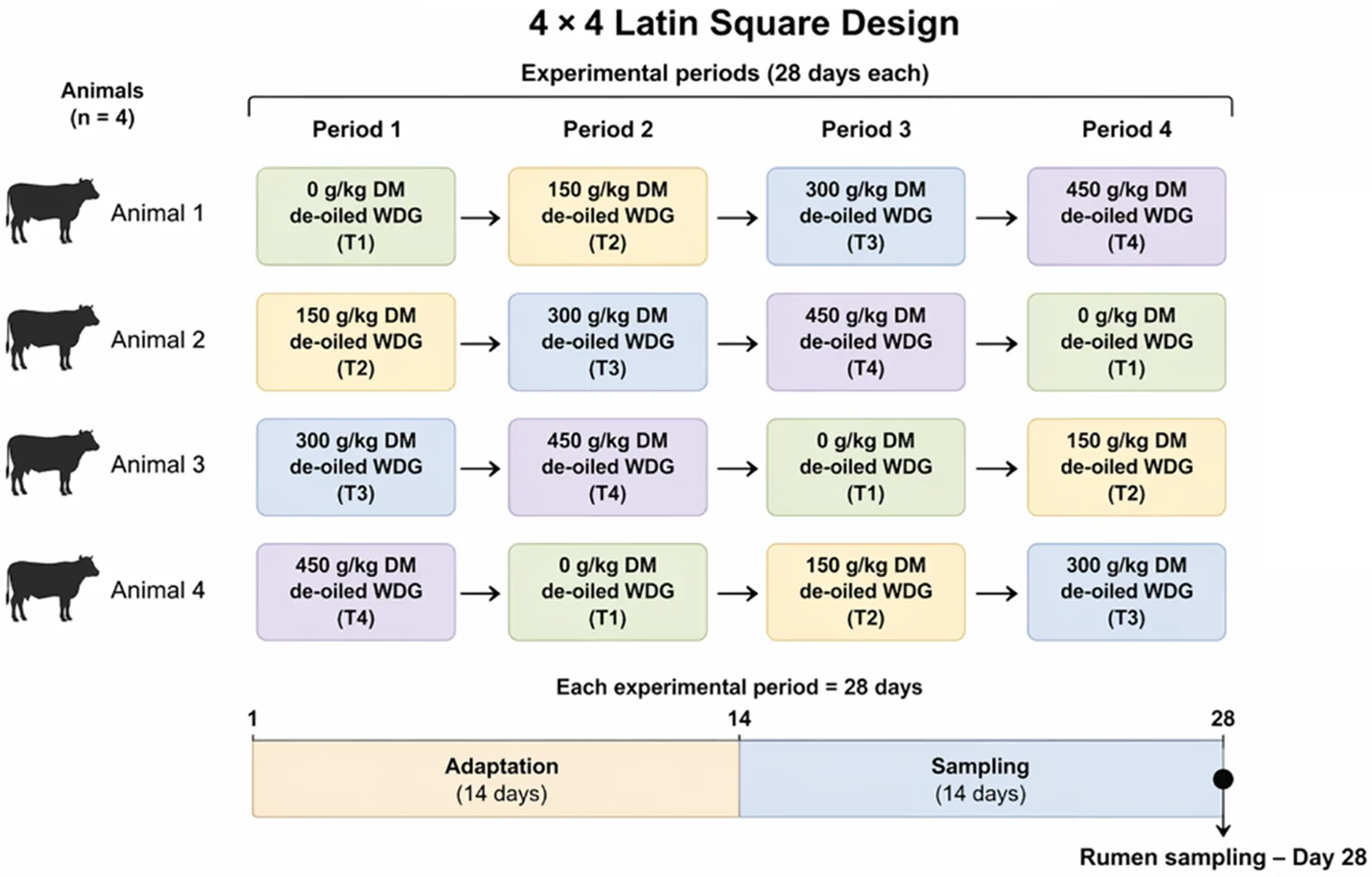
Schematic representation of the 4 × 4 Latin square design evaluating increasing levels of de-oiled WDG (0, 150, 300, and 450 g/kg DM) in Nellore bulls

Four ruminally cannulated Nellore bulls (body weight = 453 ± 32 kg) were housed in individual concrete pens and assigned to four dietary treatments containing increasing inclusion levels of de-oiled WDG (0, 150, 300, and 450 g/kg of DM). Prior to the experiment, animals were maintained on pasture under a maintenance feeding regime aimed at body weight stabilization. The experimental periods were conducted predominantly during the winter season. The experiment followed a 4 × 4 Latin square design with four 28-d experimental periods, each consisting of 14 d for dietary adaptation and 14 d for sample collection (Fig. 1). The experimental design, animal management, diet formulation, and feeding procedures were identical to those described in the companion study by Tomaz et al. (2021).

The inclusion levels of de-oiled WDG (0, 150, 300, and 450 g/kg DM) were selected to represent a practical range of incorporation in feedlot diets while generating a progressive gradient in dietary composition. In addition, the use of four levels permitted the assessment of both linear and non-linear effects of de-oiled WDG inclusion on ruminal microbial parameters.

The de-oiled WDG used in this study was obtained from a commercial ethanol plant (São Francisco Plant, Quirinópolis, Goiás, Brazil). The coproduct was compacted, stored, and ensiled in 200-L plastic containers. Before ensiling, the material was inoculated using a commercial silage inoculant with heterofermentative lactic acid bacteria (*Lactobacillus plantarum* and *Lactobacillus buchneri*; Feedtech™ Silage F600, Delaval, Tumba, Sweden) at a rate of 1 mg/kg of de-oiled WDG. The dry matter content was approximately 300 g/kg as fed. On a dry matter basis, de-oiled WDG contained 327 g/kg crude protein, 565 g/kg neutral detergent fiber, 173 g/kg acid detergent fiber, and 46 g/kg ether extract, reflecting its high protein and digestible fiber content and low starch concentration, as previously reported in the companion study by Tomaz et al. (2021).

Diets were offered as total mixed rations ad libitum twice daily, and were formulated to meet nutrient requirements for finishing beef cattle (Fox et al. 2004). The ingredient and chemical composition of the experimental diets are presented in Table 1.

**Table 1:** Chemical composition of the experimental diets

Ingredients and composition	WDG (g/kg)^1^			
	0	150	300	450
**Composition, g/kg DM**				
*Tifton* 85 Hay	42.0	42.0	42.0	42.0
Sugarcane bagasse	71.0	71.0	71.0	71.0
Ground corn	749.2	652.7	520.0	387.3
Soybean meal	103.6	47.8	29.4	11.0
Wet Distillers Grains	0	150.0	300.0	450.0
Mineral-vitamin supplement^2^	34.2	34.2	34.2	34.2
Potassium chloride	0	2.3	3.4	4.5
***Nutritional composition***,*** g/kg DM***				
Dry matter (g/kg as fed)	653.6	648.9	573.1	484.8
Ether extract	28.4	35.80	39.2	40.1
Crude protein	120.6	135.4	157.2	182.4
RDP (g/kg CP)^3^	559.5	407.3	363.1	330.8
RUP (g/kg CP)^3^	440.5	592.7	636.9	669.2
Non-fiber carbohydrates	647.5	554.3	478.3	398.4
Neutral detergent fiber	171.5	243.9	304.0	370.5
NDF_f_^4^	90.0	90.0	90.0	90.0
Calcium	7.4	7.2	7.2	7.2
Phosphorus	3.6	4.1	4.8	5.5

^1^ De-oiled corn wet distillers grains (WDG); ^2^ 18.5% Ca; 1.9% S; 1.50% Mg; 4.3% Na; 1.60% P; 1714.00ppm Zn; 1285ppm Mn; 426.00ppm Cu; 21ppm I; 5.70ppm Se; 8.5ppm Co; 285.00ppm Fe; 85700.00 UI Vit A; 11430.00 UI Vit D3; 128.00 UI Vit E; 32.5% Urea; 945.00 ppm of sodium monensin; ^3^ Rumen degradable protein and rumen undegradable protein according to NASEM (2016); ^4^ NDF from roughage

### Ruminal sampling, DNA extraction, and sequencing

For microbial community analysis, approximately 50 mL of ruminal contents, including both solid and liquid fractions, were collected by trained personnel wearing sterile gloves to minimize contamination before feeding on day 28 of the sampling period. Samples were transferred to DNA- and RNA-free tubes, rapidly cooled after collection, and stored at − 80 °C until DNA extraction and subsequent microbiome analyses.

Genomic DNA was extracted from ruminal samples following the protocols described by Weimer et al. (2010) and Pinto et al. (2020), using a bead-beating procedure combined with phenol: chloroform: isoamyl alcohol extraction.

Briefly, after thawing, 10 mL of ruminal contents were transferred to a stomacher bag and homogenized with 40 mL of DNA extraction buffer. An aliquot of the recombined sample was subjected to mechanical disruption using glass beads (Weimer et al. 2010), followed by organic solvent extraction. The extracted DNA was resuspended in 10 mM Tris-HCl containing 1 mM EDTA (pH 8.0), quantified fluorometrically using a Qubit system (Invitrogen, Carlsbad, CA, USA), and stored at − 80 °C until library preparation. Before PCR amplification, DNA samples were diluted to 10 ng/µL to ensure a minimum input of 50 ng per reaction.

Amplification of bacterial DNA targeted the V4 hypervariable region of the 16S rRNA gene using universal primers (F: GTGCCAGCMGCCGCGGTAA; R: GGACTACHVGGGTWTCTAAT), as described by Kozich et al. (2013), following the PCR conditions outlined by Pinto et al. (2020). Primers included sample-specific barcodes and Illumina-compatible adapters (F: TGATACGGCGACCACCGAGATCTACAC; R: CAAGCAGAAGACGGCATACGAGAT) to allow multiplex sequencing. Each PCR reaction contained 50 ng of template DNA, 0.4 µM of each primer, 12.5 µL of 2× Hot Start Ready Mix (KAPA Biosystems, Wilmington, MA, USA), and nuclease-free water to a final volume of 25 µL. Thermal cycling conditions consisted of an initial denaturation at 95 °C for 3 min, followed by 25 cycles of denaturation at 95 °C for 30 s, annealing at 55 °C for 30 s, extension at 72 °C for 30 s, and a final extension at 72 °C for 5 min.

PCR products were resolved on low-melt agarose gels stained with 6× Orange loading dye, and bands of approximately 380 bp were excised for purification and sequencing. Libraries were sequenced on an Illumina MiSeq platform (San Diego, CA, USA) using MiSeq v4 chemistry, with 5% PhiX included as an internal control (Dill-McFarland et al. 2017). Raw sequences were demultiplexed based on sample-specific indices and deposited in the NCBI Short Read Archive under BioProject accession PRJNA1397479.

Microbial data were analyzed within the framework of a 4 × 4 Latin square design, considering animal and period as random effects and dietary treatment as a fixed effect. The experimental unit was the individual animal within each period. Alpha diversity indices were analyzed using ANOVA, whereas beta diversity was assessed using PERMANOVA based on dissimilarity matrices, as described below.

Sequence processing and quality control were performed using Mothur (v. 1.41.1) (Schloss et al. 2009). Non-bacterial sequences were removed according to the method described by Pinto et al. (2020). Reads were aligned against the SILVA 16S rRNA reference database (v138), pre-clustered (diffs = 2) to minimize sequencing errors, and screened for chimeras using UCHIME (Edgar, 2013). Taxonomic classification was performed using the GreenGenes database (DeSantis et al. 2006), with an 80% bootstrap confidence threshold. Although this database is no longer actively maintained, it was applied consistently across all samples and was considered adequate for treatment-based comparisons. Sequences classified as cyanobacteria, mitochondria, Eukarya, or Archaea were excluded, and unique reads were removed to improve computational efficiency.

Operational taxonomic units (OTUs) were defined at 97% sequence similarity. Sequencing depth adequacy was confirmed using Good’s coverage index, with a minimum cutoff of 0.95. OTU tables were normalized to 10,000 sequences per sample before downstream analyses. Alpha diversity was evaluated using Chao1 richness and Shannon diversity indices. Diversity metrics were analyzed using a mixed model fitted in R, with dietary treatment as a fixed effect and animal and period as random effects, consistent with the 4 × 4 Latin square design. Beta diversity was examined using non-metric multidimensional scaling (NMDS) based on Bray–Curtis dissimilarity. Differences in community structure were tested using permutational multivariate analysis of variance (PERMANOVA; vegan package v2.5-2), with pairwise comparisons adjusted for false discovery rate (FDR). Relative abundances of bacterial phyla and OTUs were compared among treatments using Kruskal–Wallis tests. A total of 91 OTUs were evaluated, and all resulting P-values were adjusted for multiple testing using the Benjamini–Hochberg false discovery rate (FDR) procedure. OTU-level analyses were considered exploratory. The SIMPER procedure was applied to identify taxa contributing most to observed differences among treatments.

## Results

**Fig. 2:**
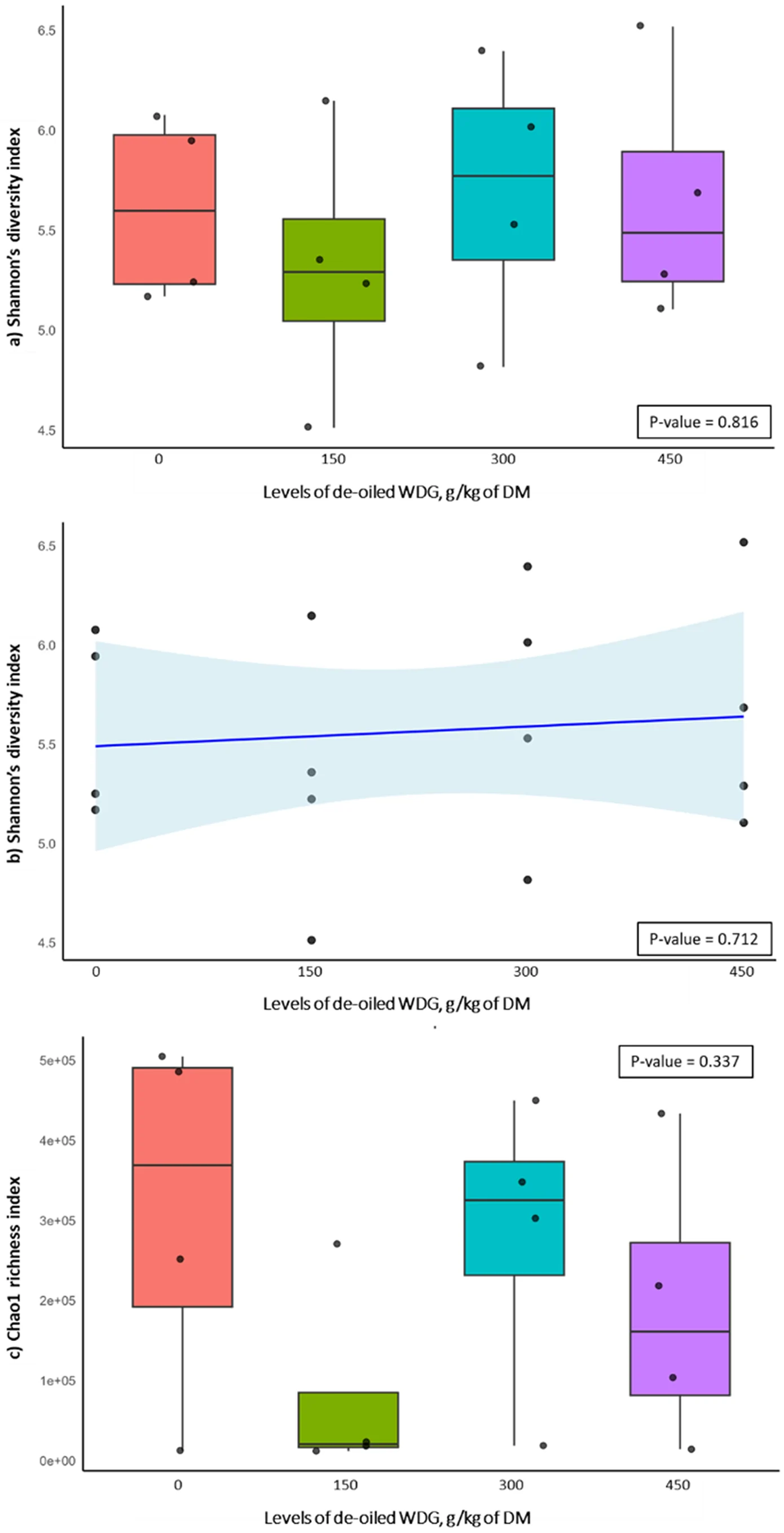
Ruminal bacterial alpha diversity in Nellore bulls fed diets containing increasing levels of de-oiled WDG (0, 150, 300, and 450 g/kg DM). (**A**) Shannon diversity index across treatments. (**B**) Relationship between dietary inclusion level of de-oiled WDG (% of diet DM) and Shannon diversity index. (**C**) Bacterial richness estimated by the Chao1 index across treatments. In boxplots (**A** and **C**), boxes represent the interquartile range, horizontal lines indicate the median, whiskers denote minimum and maximum values, and dots represent individual observations. In panel (**B**), points represent individual observations, the solid line indicates the fitted linear regression, and the shaded area represents the 95% confidence interval

Ruminal bacterial alpha diversity, evaluated using the Shannon index, was not affected by increasing levels of de-oiled WDG in the diet (*P* = 0.816; Fig. 2a). Shannon diversity indices were comparable among treatments, showing substantial overlap and no clear dose-dependent response to increasing inclusion levels, which indicates a broadly similar microbial community diversity across all dietary treatments.

When ruminal bacterial alpha diversity was evaluated as a continuous function of dietary de-oiled WDG inclusion, Shannon diversity was not linearly associated with inclusion level (*P* = 0.712; Fig. 2b). Considerable dispersion of individual observations around the fitted regression line was observed, indicating substantial inter-animal variability and the absence of a linear relationship between de-oiled WDG level and overall bacterial diversity.

Estimated ruminal bacterial richness, assessed using the Chao1 index, was not affected by increasing levels of de-oiled WDG in the diet (*P* = 0.337; Fig. 2c). Although numerical differences in Chao1 values were observed among treatments, richness estimates showed considerable convergence and high within-treatment variability, indicating comparable bacterial richness across dietary inclusion levels.

**Fig. 3:**
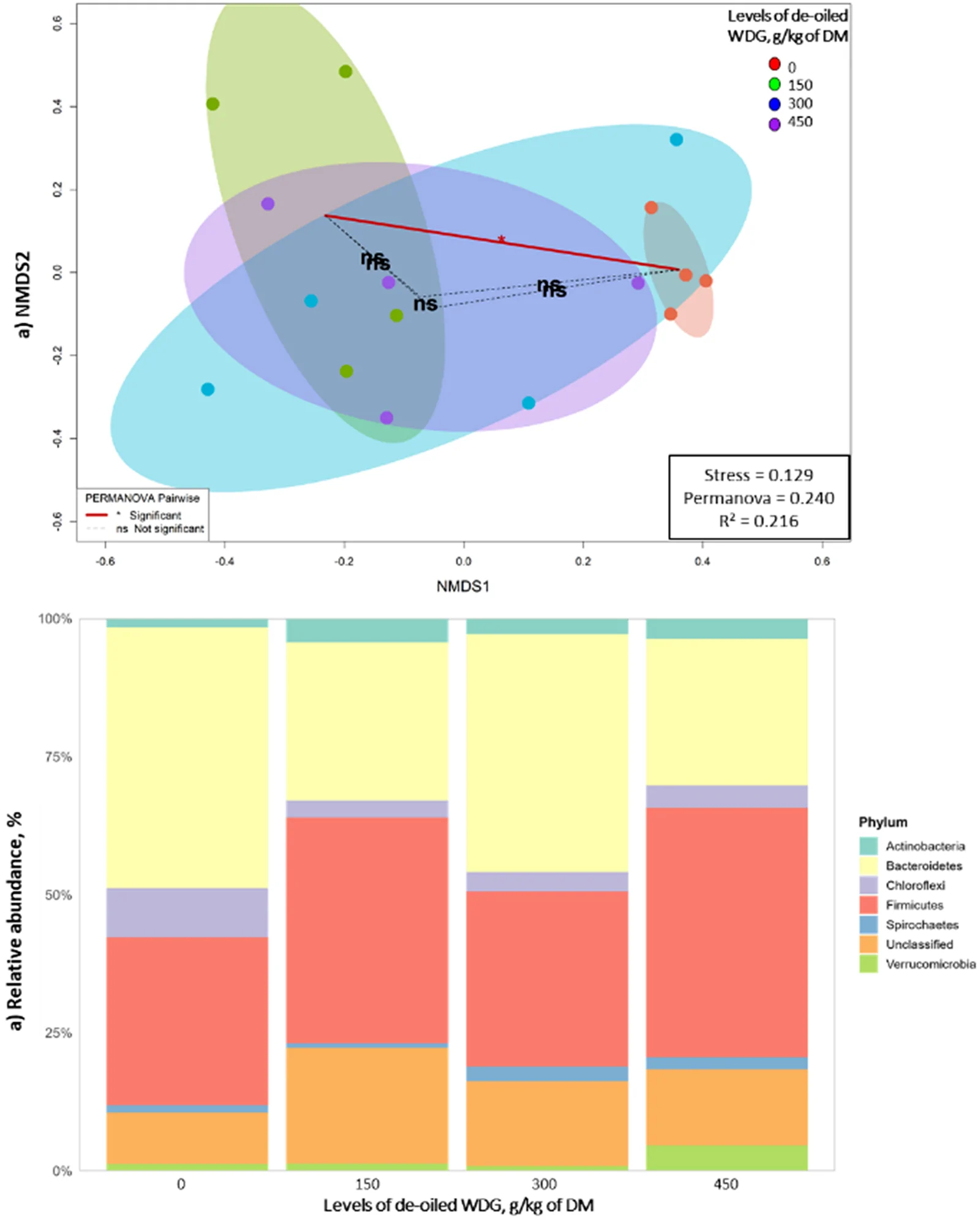
Ruminal bacterial community composition in Nellore bulls fed diets containing increasing levels of de-oiled WDG (0, 150, 300, and 450 g/kg DM). (**A**) Non-metric multidimensional scaling (NMDS) ordination based on Bray–Curtis dissimilarity. Each point represents an individual sample, and ellipses indicate the 95% confidence intervals for each dietary treatment. Community dissimilarity was evaluated by PERMANOVA (*P* = 0.262; R² = 0.216), and the stress value was 0.129. (**B**) Relative abundance of the seven most abundant bacterial phyla, expressed as percentages of total sequences. Differences among dietary treatments were evaluated using the Kruskal–Wallis test with false discovery rate (FDR) correction

Ruminal bacterial community structure, assessed by Bray–Curtis dissimilarity and visualized using NMDS ordination, did not differ among dietary treatments (PERMANOVA; *P* = 0.262; R² = 0.216; Fig. 3a). A pronounced convergence among treatment groups was evident, indicating a broadly similar bacterial community composition across increasing levels of de-oiled WDG inclusion. The NMDS ordination yielded an acceptable stress value (0.129), supporting the robustness of the two-dimensional representation of community dissimilarities. Community structure based on presence–absence data, evaluated using Jaccard dissimilarity, was not affected by dietary inclusion of de-oiled WDG (PERMANOVA; *P* = 0.269; R² = 0.208; Supplement 1), corroborating the results obtained with Bray–Curtis dissimilarity.

The relative abundance of the seven most prevalent bacterial phyla in the rumen did not differ among dietary treatments (Kruskal–Wallis; FDR-adjusted *P* > 0.05 for all phyla; Fig. 3b). *Firmicutes* and *Bacteroidetes* predominated across all treatments, followed by *Actinobacteria*, *Verrucomicrobia*, *Spirochaetes*, *Chloroflexi*, and unclassified taxa. Pairwise comparisons within the most abundant phyla did not reveal differences among dietary treatments (Supplement 2).

An exploratory analysis at the OTU level identified a limited number of taxa (OTU 013, *Firmicutes*; OTU 010 *Clostridiales*; OTU 006 *Lachnospiraceae*; OTU 004, *Mogibacterium*) showing nominal differences among dietary treatments (*P* < 0.10; Supplement 3). However, none of these differences remained significant after false discovery rate correction (FDR-adjusted *P* > 0.05), indicating that dietary inclusion of de-oiled WDG did not induce consistent or statistically supported changes in individual ruminal bacterial taxa.

## Discussion

Dietary strategies in feedlot systems often aim to modulate ruminal fermentation to improve nutrient utilization and animal performance. These changes are commonly expected to affect the ruminal microbiome, particularly under high-concentrate feeding conditions (Fernando et al. 2010; Petri et al. 2013). However, the present results show that increasing inclusion of de-oiled WDG did not alter bacterial diversity, community structure, or dominant taxa. This indicates a high degree of microbial stability under the conditions evaluated.

Alpha diversity metrics, including richness and evenness, were similar across treatments. Beta diversity analyses also showed no separation among diets. These results indicate that increasing de-oiled WDG inclusion did not disrupt overall community organization. This pattern is consistent with the concept of functional redundancy, where different taxa can perform similar metabolic roles (Weimer, 2015; Moya and Ferrer, 2016). Under this framework, dietary changes may alter metabolic activity without requiring major shifts in community structure.

Previous studies support this interpretation. The ruminal microbiome can remain structurally stable across a wide range of diets, especially after adequate adaptation (Jami and Mizrahi, 2012; Henderson et al. 2015). In the present study, replacing starch-rich ingredients with de-oiled WDG likely modified substrate availability without exceeding the adaptive capacity of the microbiota.

Dietary lipid concentration increased moderately with de-oiled WDG inclusion but remained below levels known to inhibit fibrolytic bacteria. This is consistent with the absence of changes in dominant phyla such as Bacteroidetes and Firmicutes. At the same time, increases in fiber and crude protein provided additional substrates for microbial growth. These combined changes may have supported microbial activity while preserving community structure.

Studies evaluating distillers grains in cattle have reported similar patterns. Inclusion of these coproducts often results in limited changes in microbial composition, even when fermentation profiles are altered (Castillo-Lopez et al. 2017; Dankwa et al. 2021). This suggests that microbial responses are driven more by metabolic flexibility than by taxonomic shifts.

At the OTU level, a small number of low-abundance taxa showed nominal differences among treatments. However, none remained significant after FDR correction. This indicates that these variations were not consistent or biologically robust. Such patterns are common in microbiome studies and typically reflect stochastic variation rather than structured community shifts (Thorsen et al. 2016; Weiss et al. 2017). It is important to note that the differential abundance analysis relied on conventional statistical methods that do not fully account for the compositional and sparse nature of microbiome data. Therefore, these results should be interpreted with caution, particularly for low-abundance taxa.

Marked changes in ruminal microbial structure are usually observed under strong selective pressure, such as the inclusion of antimicrobials or bioactive compounds. In contrast, de-oiled WDG does not exert direct inhibitory effects on microbial populations. This likely contributed to the stability observed in the present study.

The microbiome results are consistent with findings from the companion study by Tomaz et al. (2021). In that study, de-oiled WDG improved intake, digestibility, and fermentation profiles without affecting ruminal pH. The present results indicate that these functional responses occurred without major changes in microbial composition.

Collectively, these findings align with previous evidence indicating that distillers grains can improve energy intake and feed efficiency while maintaining ruminal homeostasis when included at moderate levels in finishing diets (Castillo-Lopez et al. 2017). As discussed by Virgínio Júnior et al. (2026), the literature evaluating ruminal microbial responses to distillers grains remains limited and heterogeneous, mainly due to differences in coproduct type, inclusion level, and experimental approach. In this context, reduced-oil and/or de-oiled products have been suggested to cause less disturbance to ruminal fermentation than conventional DDGS.

Overall, increasing inclusion of de-oiled WDG up to 450 g/kg DM did not induce detectable changes in ruminal bacterial community structure. These results suggest that this coproduct can be incorporated into high-concentrate diets without detectable alterations in the ruminal bacterial community as assessed by 16S rRNA gene sequencing.

## Conclusion

The inclusion of de-oiled wet distillers grains up to 450 g/kg DM did not affect ruminal bacterial diversity or community structure in Nellore bulls. Bacterial taxonomic profiles obtained by 16S rRNA gene sequencing remained consistent across dietary treatments. Within the scope of bacterial community profiling, these results indicate that de-oiled WDG can be included in high-concentrate diets without measurable changes in the ruminal bacterial community.

## Supplementary Information

Below is the link to the electronic supplementary material.

Supplementary Material 1

## Data Availability

Upon reasonable request, the datasets of this study can be available from the corresponding author.
