# Supplementary material for: Resilience of the ruminal bacterial community to increasing inclusion of de-oiled wet distillers grains in feedlot diets
Source: Trop Anim Health Prod. 2026 Jul 20;58(7):425. doi: 10.1007/s11250-026-05242-z (PMC13385131; doi:10.1007/s11250-026-05242-z)
Supplement: Supplementary file 1 — Supplementary Material 1 [file 11250_2026_5242_MOESM1_ESM.docx]

**SUPPLEMENTARY FILES FOR:**

**Resilience of the ruminal bacterial community to increasing inclusion of de-oiled wet distillers grains in feedlot diets**

Gercino Ferreira Virginio Júnior^1^, Pedro Henrique Francisco de Torres^1^, Barbara Carolina Afonso^1^, Bernardo Carvalho Petean^1^, Johnny Maciel de Souza^1^, Mario de Beni Arrigoni^2^, Laís de Aquino Tomaz^2^, Otávio Rodrigues Machado Neto^2^, Welder Angelo Baldassini^2^, Pablo de Souza Castagnino^3^, Danilo Domingues Millen^1*^

**^*^Corresponding Author:** **Danilo D. Millen.** E-mail: [danilo.millen@unesp.br](mailto:danilo.millen@unesp.br)

*^1^ School of Agricultural and Veterinary Sciences, São Paulo State University (UNESP), Jaboticabal, São Paulo, Brazil.*

*^2^ São Paulo State University (UNESP), School of Veterinary Medicine and Animal Science, Botucatu, São Paulo, Brazil.*

*^3^Federal University of Rio Grande do Sul, Porto Alegre, Rio Grande do Sul, Brazil*


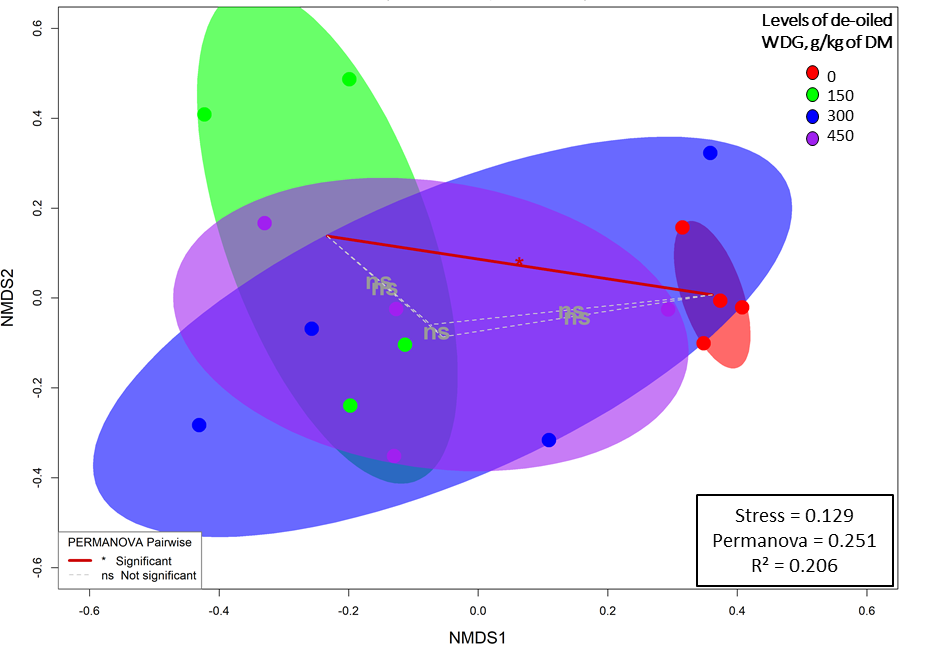


**Supplementary Figure S1.** NMDS ordination based on Jaccard dissimilarity of ruminal bacterial communities in Nellore bulls fed diets containing increasing levels of de-oiled WDG (0, 150, 300, and 450 g/kg DM). Community structure did not differ among dietary treatments (PERMANOVA; P = 0.269; R² = 0.208). The NMDS ordination presented an acceptable stress value (0.129).


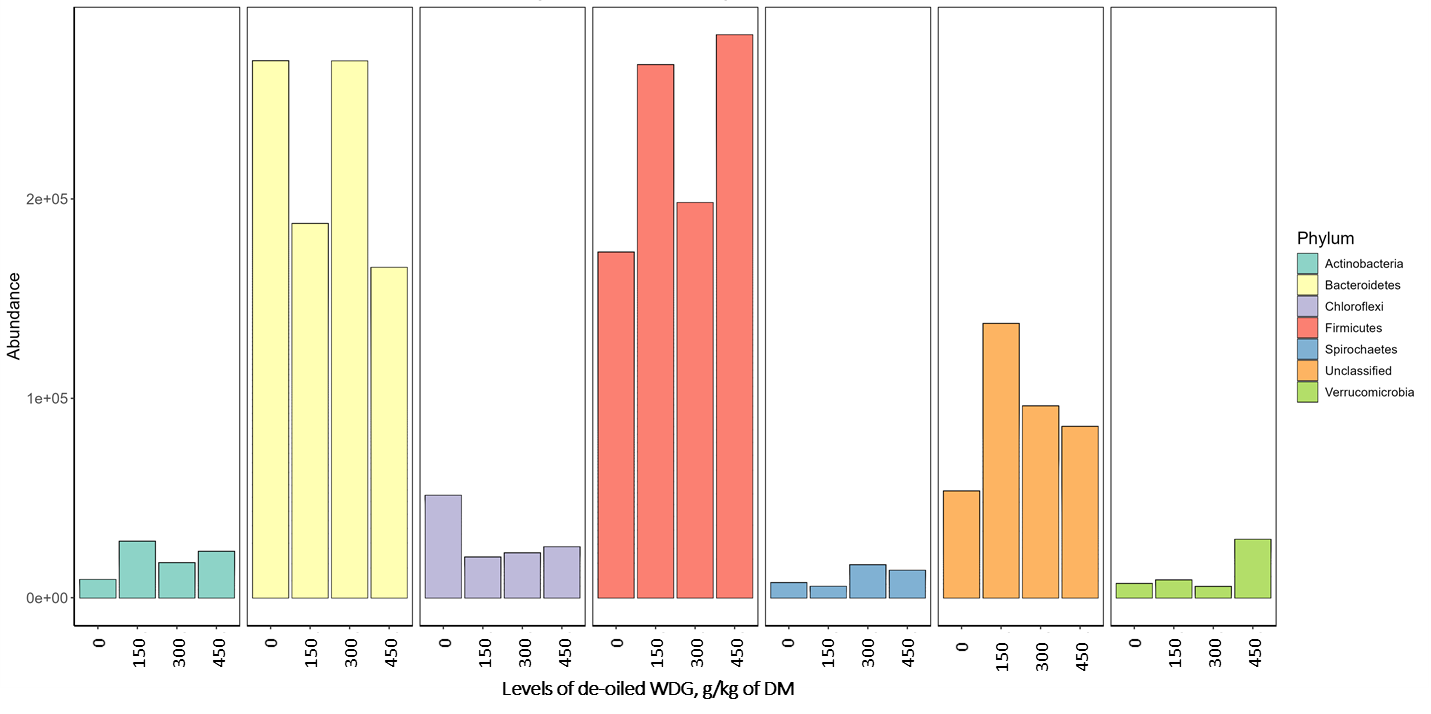


**Supplementary Figure S2.** Pairwise comparisons of phylum-level relative abundance among dietary treatments using the Wilcoxon rank-sum test. No significant differences were detected among treatments for Firmicutes or Bacteroidetes (P > 0.05 for all comparisons).


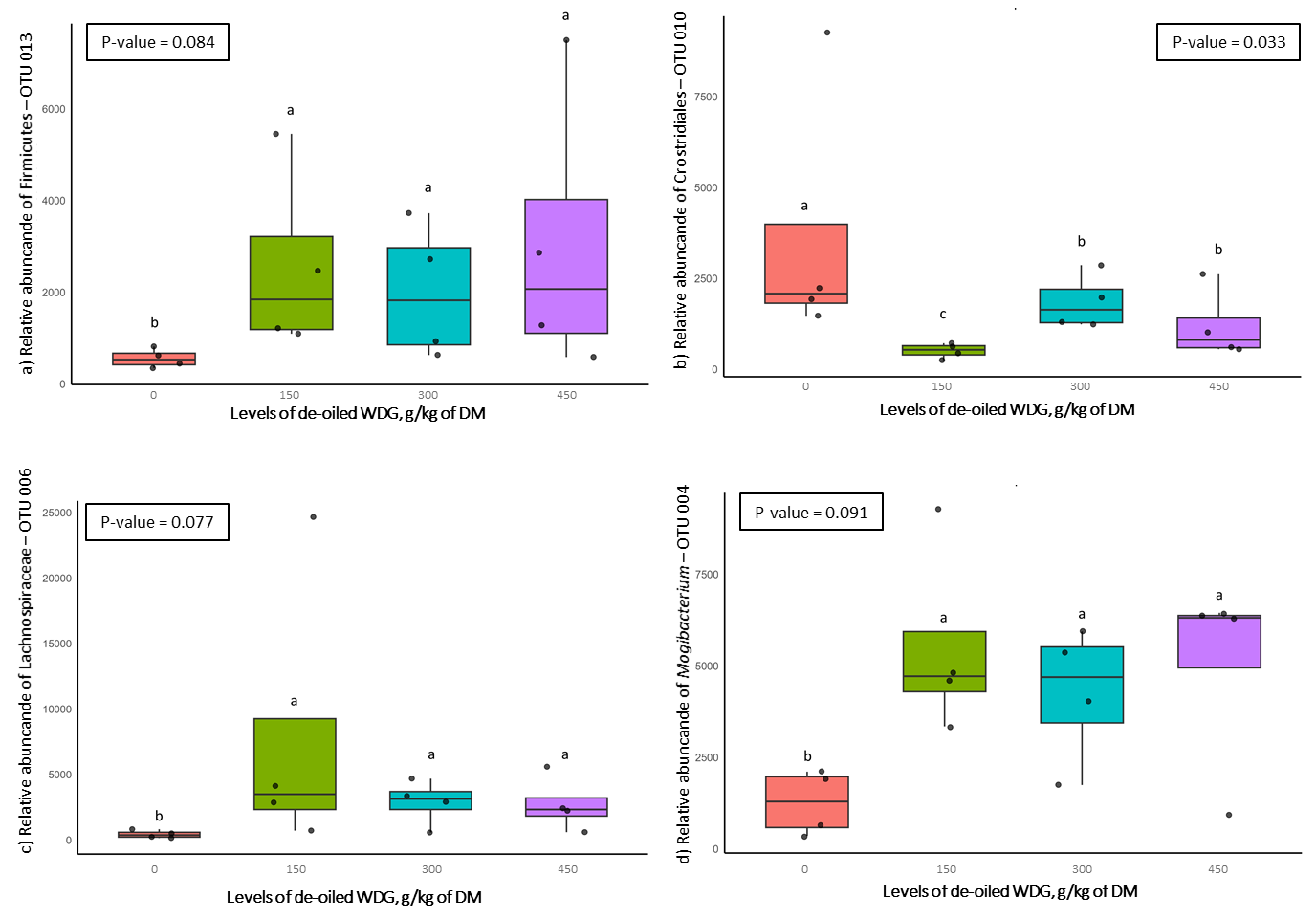


**Supplementary Figure S3.** Exploratory analysis of OTUs showing nominal differences among dietary treatments. A) Firmicutes; B) Clostridiales; C) Lachnospiraceae; D) *Mogibacterium*
